# Airway Blocks Vs LA Nebulization- An interventional trial for Awake Fiberoptic Bronchoscope assisted Nasotracheal Intubation in Oral Malignancies

**DOI:** 10.31557/APJCP.2020.21.12.3613

**Published:** 2020-12

**Authors:** Gajanan Chavan, Aparna Upadhye Chavan, Shraddha Patel, Vaibhav Anjankar, Prafulla Gaikwad

**Affiliations:** 1 *Department of Emergency Medicine, Jawaharlal Nehru Medical College, Sawangi (Meghe), Wardha, India. *; 2 *Jawaharlal Nehru Medical College, Sawangi (Meghe), Wardha, India. *; 3 *Department of Oral Medicine and Radiology, Sharad Pawar Dental College, Sawangi (Meghe), Wardha, India. *; 4 *Department of Anatomy, Jawaharlal Nehru Medical College, Sawangi (Meghe), Wardha, India. *; 5 *Department of Oral & Maxillofacial Surgery, Sharad Pawar Dental College, Sawangi (Meghe), Wardha, India. *

**Keywords:** Airway, intubation, airway blocks, superior laryngeal nerve blocks, nebulization, fiberoptic bronchoscope

## Abstract

**Background:**

Patients with intra-oral malignancies warrants use of awake Fiberoptic assisted naso-thracheal intubation to secure an airway due to multiple risk factors leading to anticipated difficult airway. Different techniques such as airway blocks, local anesthesia (LA) gargles, spray, nebulization and mild sedation are in practice to improve the success rate of fiberoptic assisted intubation.

**Methods:**

Sixty patients of ASA I and II with Mallampatti score 3 and above, posted for Commando operations were enrolled in this study and were divided into 2 groups. Group AB (Airway Block, n=30) were given Superior laryngeal nerve block bilaterally and recurrent laryngeal nerve block transtracheally with Inj 2% Lignocaine. Second Group AN (Airway Nebulization, n=30) patients airway was nebulized with 4% Lignocaine with ultrasonic nebulizer. After confirmation of satisfactory anesthesia clinically Fiber-optic assisted naso-tracheal intubation was attempted. Hemodynamic monitoring, total time taken for intubation, patients comfort and any complications occurred were noted. Statistical Analysis– All the observed values were tabulated and analyzed using software SPSS version 17.0.

**Results:**

Demography and Hemodynamic observations were comparable in the groups. The time taken for intubation, patient comfort score, intubation conditions were excellent in AB group than in group AN. Airway complications like laryngospasm and cough were noted in AN Group.

**Conclusions:**

Judicial use of combined Airway blocks such as Bilateral Superior and trans-tracheal recurrent laryngeal nerve blocks could facilitate a successful fiber-optic assisted awake naso-tracheal intubation in anticipated difficult intubation with negligible complications.

## Introduction

Consumption of the Tobacco and Betel nuts is a very rampant habit and is a common cause for Oral malignancies in India (Shah and Gil, 2009; Sankaranarayanam, 1990). Radical neck dissection (Commando Surgery) along with reconstruction is the most commonly performed surgery in these patients (Shah JP and Gil, 2009). These patients usually come in late stage and poses a common concerns to Anesthesiologist in the terms of difficult airway firstly because of tumor itself, restricted mouth opening and finally decreased inter incisor gap. Furthermore, use of Radiotherapy or chemotherapy as primary treatment could lead to more restrictions (Hancock et al., 2003).

Establishing a patent airway for General anesthesia in these patients is always challenging for anesthesiologists and risky to the patient (Benumof L, 1991; Barash et al., 2006). The Competent authorities and many studies have suggested the fibreoptic assisted intubation in anticipated difficult intubation, to avoid the life-threatening “can’t intubate, can’t ventilate scenario” (Wilson et al., 1988). Naso-tracheal intubation provides the Oncosurgeon a optimal operating conditions for mouth, pharynx, larynx and neck surgeries (Hall et al., 2003).

An idea of being awake and naso-tracheal manipulations is highly freighting in the psychologically unprepared patients, hence, sufficient time and effort must be spent to prepare the patients both mentally and by using different pharmacological modalities. Treating anesthesiologist should initiate and prepare the patients by explaining procedure in details in their mother tongue (Ramkumar 2011; Pani et al., 2009). Judicious use of Sedative and opiods should be practiced to make patient comfortable, so that the airway remains patent (Simmons et al., 2002). Once the patient understands the importance of being cooperative during the procedure the anesthesiologist wins the main portion of the battle (Farsad et al., 1978).

Multiple studies have reported an excellent hemodynamic stability with Fibreoptic Intubation (FOI) (Ovassapian et al., 1983; Sutherland and Williams, 1986; Hawkyard et al., 1992) performed with mild sedation and local anesthetic combined. Anxiety may lead to undesirable stimulation of patients sympathetic and parasympathetic systems which may lead to elevation of heart rate and blood pressure, along with troublesome excessive secretions and enhanced protective reflexes, making intubation near impossible. Knowledge of innervations and anatomy of the airway to be blocked are essential to achieve the desired effects (Pani and Rath, 2009; Simmons and Schleich, 2002). Several efficient topical and regional techniques are being used to subdue these unwanted reflexes by reducing sensations at specific regions that have to be encountered during intubation (Walsh and Shortena,1998). Topical anesthesia by Local anesthetic can be achieved by different ways such as, spray, nebulisation, atomization, gargles and topicalization (Walsh and Shortena, 1998). Ultrasonic nebulizer have less potential to cause the systemic toxicity of LA as it produces fine mist with low dose of the medications and uniformly distributes it through the airway even beyond the glottis (British Thoracic Society Bronchoscopy Guidelines Committee, 2001). However, the effect could be spotty and if the dose exceeds the recommended range may lead to systemic toxicity in these already compromised patients. Even in the sufficiently sedated patients, laryngospasm could develop due to inadequate topical anesthesia which leads to the loss of airway patency during FOB manipulations (Mcguire and El-Beheiri, 1999).

Airway blocks, on the other hand, are considered technically more difficult to perform and generally carry a higher risk of complications including bleeding, nerve damage, and intravascular injection, but, if performed by the experienced anesthesiologist it could be very useful to achieve the excellent airway anesthesia to achieve the FOI (Simmons and Schleich, 2002). To provide complete airway anesthesia, it is advisable to block the glossopharyngeal nerve (GPN), superior laryngeal nerve (SLN) bilaterally and transtracheal injection for Recurrent laryngeal nerve. Glassopharyngeal nerve can be blocked intra and extra orally but are associated with complications and it is advocated to avoid the later approach due to close proximity of the Vagus nerve (Tatjana Stopar Pintarič, 2016).

Insufficient availability of the scientific research on these techniques motivated us to design this randomized, interventional study to assess and compare the efficacy of easy to perform airway blocks with conventional spray technique to achieve upper airway anesthesia for awake FOB guided naso-tracheal intubation.

*Objectives*

1. To Check efficacy of airway blocks (AB) to achieve upper airway anesthesia for awake FOB guided naso-tracheal intubation.

2. To Check efficacy of conventional spray technique Airway Nebulization (AN) to achieve upper airway anesthesia for awake FOB guided naso-tracheal intubation.

3. To compare the efficacy of easy to perform airway blocks with conventional spray technique to achieve upper airway anesthesia for awake FOB guided naso-tracheal intubation.

## Materials and Methods

After approval of the protocol by the Institutional Ethics Committee, this interventional, crossover study was conducted at Oncosurgery OT of medical college and Hospital in India during April 2017-April 2018.

The sample size of total 60 willing and cooperative adults of age 18-80 years of ASA (American Society of Anesthesiologist) status I-II having intraoral malignancies posted for wide local excision and neck dissection were recruited for the study by taking in consideration of Level of Significance. Non-Willing, Un-cooperative, allergic to Local Anesthetic, Heamatological disorders, psychiatric ailements and patients with malignancies involving posterior third of tounge, tonsils or Larynx were excluded from the study.

A through preoperative evaluation including a complete airway evaluation (Mouth opening, Mallampati grade, thyromental distance (TM) and neck movements) was performed. Standard fasting guidelines and anti-aspiration prophylaxis with Inj. Ranitidine 50mg were prescribed. The patients were explained in details about the awake FOB (Fiberoptic) guided intubation in the pre-anesthetic evaluation in their mother tongue and informed consent were obtained.

All the study subjects were given Inj. Glycopyrrolate 0.2mg IV in receiving area before shifting to OT through newly established iv line with 18G canula. Inside the OT, standard monitoring including 5lead Electrocardiogram (ECG), non-invasive blood pressure pulse oximeter and EtCO 2 by placing sensor inside the oxygen mask were attached to the patients. Inj. Midazolam 1mg and Inj.Fentanyl 50mcg were given IV after recording the base line vitals.

The patients were randomly allocated into two groups Airway block (AB) and Airway Nebulization (AN). Randomization was done using computer generated closed envelope method. All the patients nostrils were instilled with Xylometazoline 1% (2-3) nasal drops and then gauge soaked in 4% (5ml) was placed in the nostrils for 5minutes, preferred nostril for FOB intubation was progressively dilated with silicone nasopharyngeal airway starting 6.5 upto 7.5 mm smeared with 2% lignocaine jelly.

Group AB (n- 30) were given bilateral Superior Laryngeal Nerve block with 2% plain Xylocaine (2 ml each) and transtracheal instillation (Recurrent laryngeal nerve) of 2% Xylocaine (2 ml). Six minutes wait was done. Hoarseness of voice was taken as adequate effect.

Group AN (n-30) received 10 ml of 4% Lignocaine nebulization by ultrasonic nebulizer (Omran, NE-U780) for 6 minutes.

Fibro Optic Bronchoscope (Olympus LF-DP, Tokyo, Japan) was loaded with flexo-metalic endotracheal tube 7.5mm for female and 8.0 mm for male patients.

Airway anesthesia was performed by experienced anesthesiologists in managing difficult airways. A dedicated anesthesia technician recorded all the events with stopwatch during the procedure. Time taken for intubation was from nostril up to 2cm below vocal cords in both the groups. Patients were assessed for Intubation score during the passage of FOB and patients comfort score (Table 1) (Chatrath et al., 2016) and any complications during the procedure were noted. Any sign of LAST such as ECG changes, neurological symptoms were also looked for.

Oxygenation was done continuously by venti mask with 4liter flow; mask was modified by cutting a hole at the top for FOB insertion. After passage of ETT into trachea and carina under vision General Anesthesia was administered with Propofol 2ml/ kg and Rocuronium 0.6ml/kg IV and connected to the ventilator. Hemodynamic monitoring was concluded after 4 minutes post intubation.

Statistical Analysis: All data was tabulated and analysed using software SPSS 17.0.All continuous parametric data were compared using the unpaired Students t-Test and non-parametric data such as intubation grade and patients’ comfort score were compared using Chi-square test. Statistical significant value was considered if p< 0.05, and if p<0.001 as highly significant. Results were analyzed and compared with previous studies. Power of the study was calculated to be above 90%.

## Results

**Figure 1:**
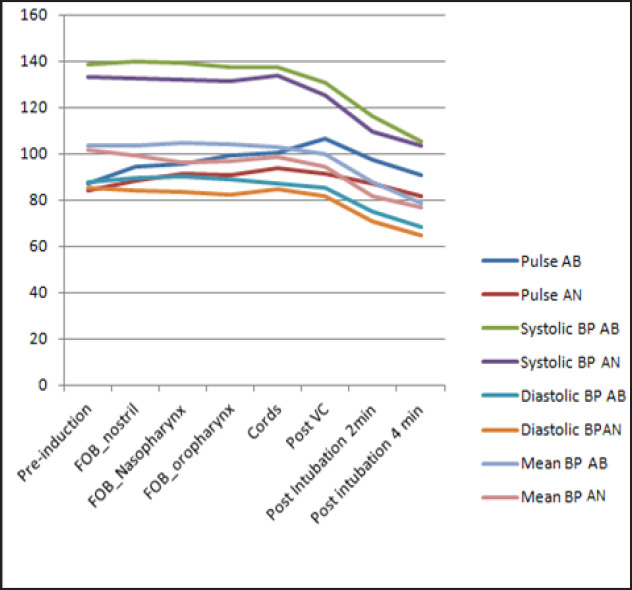
Graph Showing Haemodynamic Characteristics of the Two Groups

**Figure 2:**
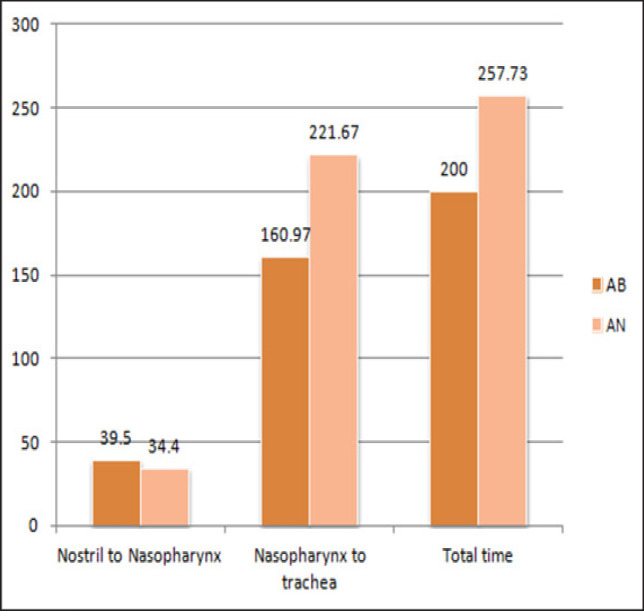
Graph Showing Mean Time Taken for Intubation

The demographic data (Table II) was comparable in two groups. There was no significant difference in heart rate (HR) and Blood pressure (BP) in groups (Figure 1). However, there was slight drop in SpO_2_ upto 95% in group AN during FOB at cords and continued to post cord position (Figure 2). All the patinents remained awake and cooperative, but two patients in AN group developed Laryngospasm and to be intubated with administration of GA (T2).

The total time taken for intubation was 200 (156.48-261.59) sec in AB group which was significantly less than AN group 257.73(212.97-304.43) sec. (Table II and figure II). Intubation score was optimal in 90% of cases in AB group as compared to 36% in AN group (table III). Three Point patient comforts during FOB passage through vocal cords was statistically highly significant in AB as compared to AN group with p< 0.01 (Table III), similarly, 5 point score in no reaction was excellent in AB group (Table III).

**Table 1:** Intubation Score and Patient Comfort Score Assessed during the Passage of Fibrescope to Assess the Efficacy of Airway Block

Intubation Score	A.Vocal cord movements	
	Open	
	Moving	
	Closing	
	Closed	
	B.Coughing	
	None	
	Slight	
	Moderate	
	Severe	
	C.Limb movement	
	None	
	Slight	
	Moderate	
	Severe	
Patient comfort score	5point fiberoptic intubation comfort score No reaction Slight grimacing Heavy grimacing Verbal objection Defensive movement of head or hands	3point score immediately after orotracheal intubation Cooperative Restless/minimal resistance Severe resistance

**Table 2:** Demographic Data, Mallampatti Score, Mouth Opening, Thyromental Distance, Neck Movements, Complications and Time Taken for Intubation

Age (mean)	Airway Nebulization (AN)		Airway Block ( AB)	
	47.03±10.4		46.83±8.0	
Sex	Male	Female	Male	Female
	23	7	25	5
Mallampatti Score	AN		AB	
	III	IV	III	IV
	19	11	15	15
Mouth Opening	AN		AB	
Mean ± SD	1.13 ± 0.35		1.03 ± 0.46	
Thyromental Distance	AN		AB	
Mean ± SD	3.87 ± 0.63		3.99 ± 0.60	
Neck Movements	Normal	Decrease	Normal	Decrease
	27	3	30	0
Complications	AN		AB	
Bradycardia	2		0	
Tachycardia	10		2	
Hypertension	4		2	
Laryngospasm	2		0	
Time taken for Intubation in Seconds	AN		AB	
Nostril- Nasopharynx	35.59 ± 16.65		39.5 ± 20.46	
Nasopharynx Trachea	229.31 ± 111.630		160.96 ± 142.48	
Total time taken for Intubation	266.62 ± 115.86		200 ± 146.35	

**Table 3:** Grades of Intubating Conditions, 3 and 5 Point Intubation Score Assessed During FOB Passage

Intubating Conditions		AN	AB
Optimal		11	27
Suboptimal		16	3
Difficult		1	0
Failed		2	0
Optimal Vs other groups- Chi square value 7.9, p value <0.04			
3 point comfort score		AN	AB
Vocal cord movements	Open	3	23
	Moving	25	6
	Closed	2	1
Coughing	Slight	14	7
	None	10	23
	Moderate	3	0
	Severe	3	0
	Slight	9	7
Limb Movement	Moderate	14	2
	None	7	21
In vocal cord open vs other - Chi square value 27.1 (p value <0.001) In coughing none vs other groups- Chi square value 11.38 (p value <0.01) In Limb movement None vs others- Chi square value 13.12 (p value <0.001)			
5 Points	AN	AB	
No Reaction	2	17	
Slight Grimacing	23	11	
Heavy Grimacing	2	2	
Verbal Objection	1	0	
Defensive Head & Hand Movements	2	0	
No reaction vs others - Chi square value 17.33 ( p value <0.001)			

## Discussion

Awake FOB assisted tracheal intubation was practiced for the first time by Murphy in 1967 and is being extensively authenticated by different authors in management of difficult intubation (Edens and Sia, 1981, Monrigal et al., 1991; Nakayama et al., (1992). However very limited studies are available in literature like the present study. 

Patients with oral malignancies always falls in the category of anticipated difficult airway or intubation. FOB assisted naso-tracheal intubation in these cases is safest method to achieve it (Popat, 2003). Using conventional GA in these patients is against the prescribed protocol of difficult airway algorithm which includes call for help may not be applicable as time won’t be there (Lallo et al., 2009). 

An awake FOB intubation allows the patient to maintain the tone of airway hence provides a degree of safety margin which is lost if the patient is given GA (Benumof, 1991; Johnson and Roberts, 1989). Awake FOB intubation is challenging to anesthesiologist and frightening to the patient, hence, proper preoperative empathic counseling is extremely vital to succeed. Judicious use of anti-sialogouges, anxiolytics and analgesics is also mandatory (Ramkumar, 2011; Pani and Rath, 2009). Different conventional modalities to anesthetize the passage to negotiate the FBO such as, atomization, nebulisation gargles, spray and lozenges are in use. These conventional modalities does not always provide satisfactory results and are associated with drawbacks like spotty coverage, LAST and even loss of the airway, along with heightened sensitivity which can lead to complication like Laryngospasm.

In our study, the mean time taken for intubation was significantly less in AB than in AN group. Our results contradict with study done by Reasoner et al., (1995). This was probably due to supplementation RLN block, which might have improved the anesthesia. We have similar trend in mean time taken for intubation to the study conducted by Gupta et al., (2004) and Veena et al., (2016). Kundra et al., (2000) compared nebulisation of 4% lignocaine with airway blocks (RLN, SLN) and quoted higher stress, grimace scores and hemodynamic fluctuations in nebulisation group during FOB passage which corresponds to present study. Studies conducted by Gupta et al., (2014), Ovassapian et al., (1983), Graham et al., (1992) and Trivedi and Patil (2009) have similar outcome as of our study in the terms of intubation score and patients comfort in their nerve block groups. Complications observed in present study were also mentioned in different protocols but statistical significance could not be established.

Bilateral SLN and trans-tracheal RLN block, adequate mental and pharmacological preparation of upper airway and empathetic attitude towards patient aids in achieving FOB assisted intubation in awake patients as compared to only spray technique. 

*Shortfall of study*

Our study have short fall of minimal bias as it is not a blind study and secondly our institution is not equipped with facility to measure serum lignocaine levels but clinically no patients have signs or symptoms of LAST.

In conclusion, combined airway nerve blocks provides excellent intubating conditions for fiberoptic assisted naso-tracheal intubations with desired hemodynamic stability, maximum patient comfort and minimum required sedation.
